# Crystal structure of [1,3-bis­(di­phenyl­phosphan­yl)propane-κ^2^ *P*,*P*′](*N*,*N*′-di­methyl­thio­urea-κ*S*)(thio­cyanato-κ*N*)copper(I)

**DOI:** 10.1107/S2056989015002479

**Published:** 2015-02-11

**Authors:** Yupa Wattanakanjana, Arunpatcha Nimthong-Roldán, Janejira Ratthiwan

**Affiliations:** a Department of Chemistry, Faculty of Science, Prince of Songkla University, Hat Yai, Songkhla 90112, Thailand; b Department of Chemistry, Youngstown State University, 1 University Plaza, 44555 Youngstown, OH, USA

**Keywords:** crystal structure, *N*,*N*′-di­methyl­thio­urea, copper(I) complex, hydrogen bonding

## Abstract

The asymmetric unit of the title compound, [Cu(NCS)(C_3_H_8_N_2_S)(C_27_H_26_P_2_)], contains two independent mononuclear complex mol­ecules. In each, the Cu^I^ ion exhibits a distorted tetra­hedral geometry by coordination with two P atoms from one 1,3-bis(diphenylphosphino)propane (dppm) ligand, one terminal S atom of one *N*,*N*′-di­methyl­thio­urea (dmtu) ligand and one terminal N atom of the thio­cyanato ligand. The dppp ligand is involved in a bidentate coordination mode with the Cu^I^ ion, forming a six-membered CuP_2_C_3_ ring. In both mol­ecules, the coordination of the dmtu ligand is further stabilized by an intra­molecular N—H⋯N hydrogen bond with an *S*(6) graph-set motif. In the crystal, mol­ecules are linked by N—H⋯S hydrogen bonds forming a zigzag chain along the *a*-axis direction. In one independent mol­ecule, one of the phenyl rings of the dppp ligand is disordered over two sites with refined occupancies 0.639 (11):0.361 (11) and this corresponds with a mutual disorder of the dmtu ligand in the other independent mol­ecule giving the same ratio of refined occupancies. The structure was refined as a two-component inversion twin.

## Related literature   

For applications of thio­urea, thio­urea derivatives and their complexes, see: Chen *et al.* (2009 ▸); Isab *et al.* (2010 ▸); Saeed *et al.* (2010 ▸).
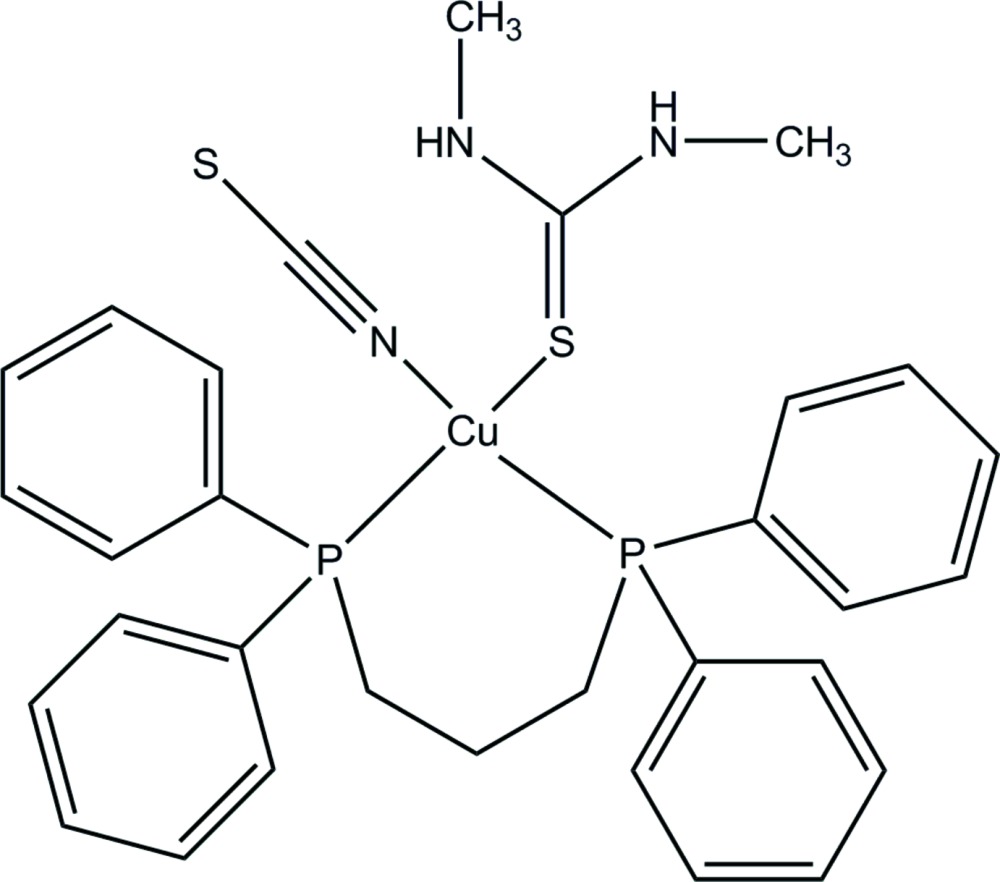


## Experimental   

### Crystal data  

[Cu(NCS)(C_3_H_8_N_2_S)(C_27_H_26_P_2_)]
*M* *_r_* = 638.21
Monoclinic, 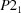

*a* = 9.9727 (15) Å
*b* = 31.971 (5) Å
*c* = 10.2162 (15) Å
β = 110.022 (2)°
*V* = 3060.4 (8) Å^3^
*Z* = 4
Mo *K*α radiation
μ = 0.98 mm^−1^
*T* = 100 K
0.44 × 0.42 × 0.26 mm

### Data collection  

Bruker APEXII CCD diffractometer
Absorption correction: multi-scan *SADABS* (Bruker, 2013 ▸) *T*_min_ = 0.566, *T*_max_ = 0.746
24402 measured reflections
13908 independent reflections
12457 reflections with *I* > 2σ(*I*)
*R*_int_ = 0.027

### Refinement  

*R*[*F* ^2^ > 2σ(*F* ^2^)] = 0.045
*wR*(*F* ^2^) = 0.111
*S* = 1.06
13908 reflections
744 parameters
140 restraints
H-atom parameters constrained
Δρ_max_ = 1.32 e Å^−3^
Δρ_min_ = −0.69 e Å^−3^
Absolute structure: refined as an inversion twin
Absolute structure parameter: 0.158 (14)

### 

Data collection: *APEX2* (Bruker, 2013 ▸); cell refinement: *SAINT* (Bruker, 2013 ▸); data reduction: *SAINT*; program(s) used to solve structure: *SHELXS97* (Sheldrick, 2008 ▸); program(s) used to refine structure: *SHELXL2014* (Sheldrick, 2015 ▸) and *SHELXLE* (Hübschle *et al.*, 2011 ▸); molecular graphics: *Mercury* (Macrae, 2008 ▸); software used to prepare material for publication: *SHELXL97* (Sheldrick, 2008 ▸) and *publCIF* (Westrip, 2010 ▸).

## Supplementary Material

Crystal structure: contains datablock(s) I. DOI: 10.1107/S2056989015002479/lh5751sup1.cif

Structure factors: contains datablock(s) I. DOI: 10.1107/S2056989015002479/lh5751Isup2.hkl

**Fig. 1.**
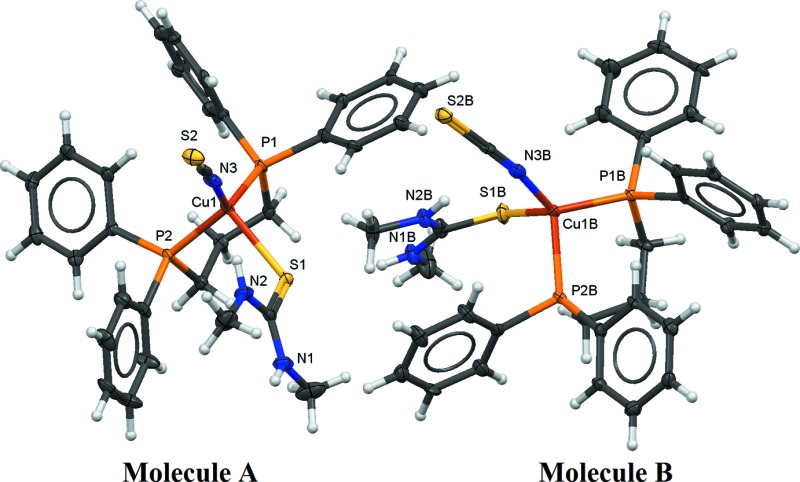
The molecular structure with displacement ellipsoids drawn at the 50% probability level. The minor component of disorder is omitted for clarity.

**Fig. 2.**
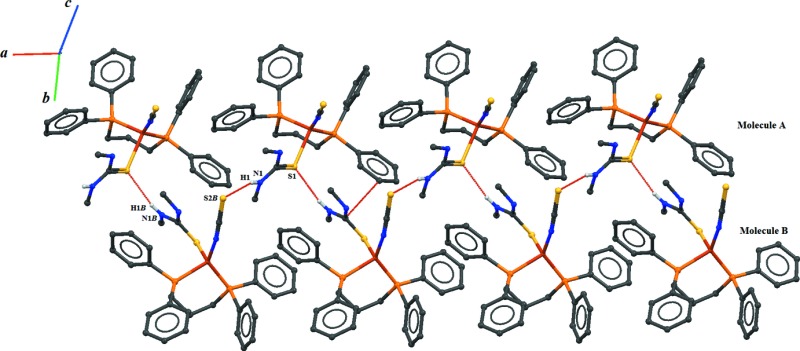
Part of the crystal structure showing intermolecular N—H···S hydrogen bonds as dashed lines, forming a zigzag chain along the a-axis.

. DOI: 10.1107/S2056989015002479/lh5751fig1.tif
The mol­ecular structure with displacement ellipsoids drawn at the 50% probability level. The minor component of disorder is omitted for clarity.

a . DOI: 10.1107/S2056989015002479/lh5751fig2.tif
Part of the crystal structure showing inter­molecular N—H⋯S hydrogen bonds as dashed lines, forming a zigzag chain along the *a*-axis.

CCDC reference: 1047497

Additional supporting information:  crystallographic information; 3D view; checkCIF report

## Figures and Tables

**Table 1:** Hydrogen-bond geometry (, )

*D*H*A*	*D*H	H*A*	*D* *A*	*D*H*A*
N1H1S2*B* ^i^	0.88	2.64	3.466(5)	158
N2H2N3	0.88	2.32	3.174(7)	165
N2*B*H2*L*N3*B*	0.88	2.43	3.286(13)	164
N1*B*H1*B*S1	0.88	2.55	3.350(13)	151
N2*C*H2*M*N3*B*	0.88	2.50	3.24(2)	142
N1*C*H1*C*S1	0.88	2.58	3.34(2)	146

Symmetry code: (i) 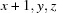.

## supplementary crystallographic information

### S1. Comment

Thio­urea and thio­urea derivatives as well as their complexes have been widely studied because of their relevance in biological systems such as anti­microbial, anti­viral and anti fungal (Chen *et al.*, 2009; Isab *et al*., 2010; Saeed *et al.*, 2010). The use of diphosphine lead to the mononuclear complex we have prepared in this study. The asymmetric unit of the title compound, [Cu(NCS)(C_3_H_8_N_2_S)(C_27_H_26_P_2_)], contains two independent mononuclear complex molecules (Fig. 1). In each, the Cu^I^ ion exhibits a distorted tetra­hedral geometry by coordination with two P atoms from one propane-1,3-bis­(di­phenyl­phosphino) (dppm) ligand, one terminal S atom of one *N*,*N*'-di­methyl­thio­urea (dmtu) ligand and one terminal N atom of thio­cyanate ligand. The dppp ligand is involved in a bidentate coordination mode with the Cu^I^ ion, forming a six-membered CuP_2_C_3_ ring. In both molecules, the coordination of the dmtu ligand is further stabilized by an intra­molecular N—H···N hydrogen bond (Table 1) with S(6) graph-set motif. In the crystal, molecules are linked by N—H···S hydrogen bonds forming a one-dimensional zigzag chain along the *a*-axis direction (see Table 1 and Fig. 2). In molecule A, one of the phenyl rings of the dppp ligand is disordered over two sites with refined occupancies 0.639 (11):0.361 (11) and this corresponds with a mutual disorder of the dmtu ligand in molecule B giving the same ratio of refined occupancies.

### S2. Experimental

Propane-1,3-bis­(di­phenyl­phosphino), dppp, (0.34 g, 0.8 mmol) was dissolved in 30 ml of aceto­nitrile at 338 K and then copperthio­cynate, CuSCN, (0.10 g, 0.8 mmol) was added. The mixture was stirred for 3 hr and then *N*,*N*′-di­methyl­thio­urea, dmtu, (0.09 g, 0.8 mmol) was added and the new reaction mixture was heated under reflux for 4 hr during which the precipitate gradually disappeared. The resulting clear solution was filtered and left to evaporate at room temperature. The crystalline complex, which deposited upon standing for several days, was filtered off and dried in vacuo.

### S2.1. Refinement

Reflections 020, 0-20, -505, -605 and 006 were affected by the beam stop and were omitted from the refinement. H atoms bonded to C and N atoms were included in calculated positions and were refined with a riding model using distances of 0.95 Å (aryl H), and U_iso_(H) = 1.2U_eq_(C); 0.98 Å (CH_3_) and U_iso_(H) = 1.5U_eq_(C); 0.88 Å (NH), and U_iso_(H) = 1.2U_eq_(N). One of phenyl rings of the dppp ligand in molecule A is mutually disordered with the dmtu of molecule B. The ADPs of the carbon and nitro­gen atoms were constrained to be identical. An enhanced rigid bond restraint was applied to all disordered atoms [RIGU in SHELXL (Sheldrick, 2008)]. To ensure satisfactory refinement the atoms of each disorder component of the phenyl ring and the directly attached carbon atoms were each restrained to lie within a common plane. The overall ratio of the two components of disorder refined to 0.639 (11) and 0.361 (11).

### Crystal data

**Table:**

[Cu(NCS)(C_3_H_8_N_2_S)(C_27_H_26_P_2_)]	*F*(000) = 1328
*M**_r_* = 638.21	*D*_x_ = 1.385 Mg m^−^^3^
Monoclinic, *P*2_1_	Mo *K*α radiation, λ = 0.71073 Å
*a* = 9.9727 (15) Å	Cell parameters from 986 reflections
*b* = 31.971 (5) Å	θ = 2.9–29.6°
*c* = 10.2162 (15) Å	µ = 0.98 mm^−^^1^
β = 110.022 (2)°	*T* = 100 K
*V* = 3060.4 (8) Å^3^	Prism, colourless
*Z* = 4	0.44 × 0.42 × 0.26 mm

### Data collection

**Table:**

Bruker APEXII CCD diffractometer	13908 independent reflections
Radiation source: fine focus sealed tube	12457 reflections with *I* > 2σ(*I*)
Graphite monochromator	*R*_int_ = 0.027
ω and φ scans	θ_max_ = 31.6°, θ_min_ = 2.1°
Absorption correction: multi-scan *SADABS* (Bruker, 2013)	*h* = −14→13
*T*_min_ = 0.566, *T*_max_ = 0.746	*k* = −45→44
24402 measured reflections	*l* = −13→12

### Refinement

**Table:**

Refinement on *F*^2^	Secondary atom site location: difference Fourier map
Least-squares matrix: full	Hydrogen site location: inferred from neighbouring sites
*R*[*F*^2^ > 2σ(*F*^2^)] = 0.045	H-atom parameters constrained
*wR*(*F*^2^) = 0.111	*w* = 1/[σ^2^(*F*_o_^2^) + (0.0442*P*)^2^ + 4.2761*P*] where *P* = (*F*_o_^2^ + 2*F*_c_^2^)/3
*S* = 1.06	(Δ/σ)_max_ = 0.001
13908 reflections	Δρ_max_ = 1.32 e Å^−^^3^
744 parameters	Δρ_min_ = −0.69 e Å^−^^3^
140 restraints	Absolute structure: Refined as an inversion twin.
Primary atom site location: structure-invariant direct methods	Absolute structure parameter: 0.158 (14)

### Special details

**Table:**

Geometry. All e.s.d.'s (except the e.s.d. in the dihedral angle between two l.s. planes) are estimated using the full covariance matrix. The cell e.s.d.'s are taken into account individually in the estimation of e.s.d.'s in distances, angles and torsion angles; correlations between e.s.d.'s in cell parameters are only used when they are defined by crystal symmetry. An approximate (isotropic) treatment of cell e.s.d.'s is used for estimating e.s.d.'s involving l.s. planes.
Refinement. Refined as a 2-component inversion twin.

### Fractional atomic coordinates and isotropic or equivalent isotropic displacement parameters (Å^2^)

**Table:**

	*x*	*y*	*z*	*U*_iso_*/*U*_eq_	Occ. (<1)
Cu1	0.23083 (6)	0.48822 (2)	0.95389 (7)	0.01440 (14)	
S1	0.29244 (14)	0.55158 (4)	0.85602 (16)	0.0173 (3)	
S2	0.24387 (16)	0.52907 (5)	1.40265 (17)	0.0247 (3)	
P1	0.05689 (13)	0.46187 (4)	0.77085 (16)	0.0139 (3)	
P2	0.41776 (13)	0.45185 (4)	0.93301 (15)	0.0128 (3)	
N1	0.5053 (5)	0.60594 (15)	0.9529 (6)	0.0244 (11)	
H1	0.5669	0.6201	1.0210	0.029*	
N2	0.4300 (5)	0.57410 (15)	1.1162 (6)	0.0244 (12)	
H2	0.3690	0.5572	1.1342	0.029*	
N3	0.2224 (5)	0.50129 (15)	1.1380 (6)	0.0201 (11)	
C1	0.4191 (5)	0.57906 (15)	0.9854 (6)	0.0167 (12)	
C2	0.5035 (9)	0.6131 (2)	0.8124 (9)	0.0425 (19)	
H2C	0.5120	0.5863	0.7693	0.064*	
H2D	0.5836	0.6313	0.8151	0.064*	
H2E	0.4135	0.6267	0.7578	0.064*	
S2B	−0.24826 (15)	0.67958 (5)	1.13983 (16)	0.0219 (3)	
C1B	−0.0620 (17)	0.6408 (6)	0.7188 (12)	0.0139 (19)	0.639 (11)
N2B	−0.0397 (12)	0.6469 (4)	0.8525 (12)	0.019 (2)	0.639 (11)
H2L	−0.1034	0.6619	0.8737	0.022*	0.639 (11)
C3B	0.0815 (14)	0.6310 (4)	0.9691 (15)	0.032 (3)	0.639 (11)
H3BA	0.0888	0.6006	0.9598	0.048*	0.639 (11)
H3BB	0.0670	0.6373	1.0571	0.048*	0.639 (11)
H3BC	0.1696	0.6443	0.9682	0.048*	0.639 (11)
N1B	0.0284 (14)	0.6167 (4)	0.6824 (12)	0.023 (2)	0.639 (11)
H1B	0.1064	0.6080	0.7480	0.027*	0.639 (11)
C2B	0.0045 (15)	0.6042 (5)	0.5409 (13)	0.042 (3)	0.639 (11)
H2F	−0.0854	0.5887	0.5050	0.063*	0.639 (11)
H2G	0.0833	0.5863	0.5381	0.063*	0.639 (11)
H2H	−0.0007	0.6291	0.4834	0.063*	0.639 (11)
C1C	−0.060 (3)	0.6427 (12)	0.751 (3)	0.0139 (19)	0.361 (11)
N2C	−0.061 (2)	0.6389 (7)	0.881 (2)	0.019 (2)	0.361 (11)
H2M	−0.1332	0.6495	0.9012	0.022*	0.361 (11)
C3C	0.053 (3)	0.6174 (7)	0.990 (3)	0.032 (3)	0.361 (11)
H3BD	0.0438	0.5871	0.9744	0.048*	0.361 (11)
H3BE	0.0473	0.6241	1.0811	0.048*	0.361 (11)
H3BF	0.1459	0.6266	0.9864	0.048*	0.361 (11)
N1C	0.045 (3)	0.6249 (8)	0.720 (2)	0.023 (2)	0.361 (11)
H1C	0.1130	0.6126	0.7876	0.027*	0.361 (11)
C2C	0.056 (3)	0.6241 (8)	0.584 (2)	0.042 (3)	0.361 (11)
H2I	−0.0264	0.6093	0.5193	0.063*	0.361 (11)
H2J	0.1439	0.6096	0.5876	0.063*	0.361 (11)
H2K	0.0580	0.6528	0.5506	0.063*	0.361 (11)
P2B	−0.08332 (14)	0.76514 (4)	0.67173 (16)	0.0140 (3)	
C3	0.5364 (7)	0.5947 (2)	1.2340 (8)	0.0344 (16)	
H3A	0.6323	0.5878	1.2343	0.052*	
H3B	0.5260	0.5851	1.3211	0.052*	
H3C	0.5224	0.6251	1.2256	0.052*	
N3B	−0.2716 (5)	0.71589 (15)	0.8829 (6)	0.0246 (12)	
C4	0.2307 (5)	0.51225 (16)	1.2477 (6)	0.0158 (12)	
C11	−0.1138 (19)	0.4900 (4)	0.698 (3)	0.0175 (17)	0.639 (11)
C12	−0.2334 (19)	0.4711 (4)	0.604 (2)	0.016 (3)	0.639 (11)
H12	−0.2279	0.4434	0.5723	0.020*	0.639 (11)
C13	−0.3634 (19)	0.4934 (4)	0.555 (2)	0.016 (3)	0.639 (11)
H13	−0.4469	0.4800	0.4949	0.019*	0.639 (11)
C14	−0.3700 (15)	0.5338 (5)	0.594 (2)	0.025 (3)	0.639 (11)
H14	−0.4554	0.5494	0.5538	0.030*	0.639 (11)
C15	−0.2522 (10)	0.5522 (3)	0.6918 (13)	0.023 (3)	0.639 (11)
H15	−0.2591	0.5798	0.7233	0.028*	0.639 (11)
C16	−0.1228 (11)	0.5302 (3)	0.7446 (13)	0.022 (3)	0.639 (11)
H16	−0.0422	0.5428	0.8119	0.027*	0.639 (11)
C11C	−0.113 (3)	0.4881 (7)	0.688 (5)	0.0175 (17)	0.361 (11)
C12C	−0.237 (3)	0.4653 (8)	0.625 (5)	0.016 (3)	0.361 (11)
H12C	−0.2342	0.4356	0.6240	0.020*	0.361 (11)
C13C	−0.367 (3)	0.4861 (8)	0.564 (5)	0.016 (3)	0.361 (11)
H13C	−0.4513	0.4704	0.5204	0.020*	0.361 (11)
C14C	−0.373 (3)	0.5281 (9)	0.567 (5)	0.025 (3)	0.361 (11)
H14C	−0.4628	0.5416	0.5295	0.030*	0.361 (11)
C15C	−0.2497 (18)	0.5521 (5)	0.623 (2)	0.023 (3)	0.361 (11)
H15C	−0.2531	0.5818	0.6161	0.028*	0.361 (11)
C16C	−0.120 (2)	0.5312 (7)	0.691 (3)	0.022 (3)	0.361 (11)
H16C	−0.0363	0.5468	0.7383	0.027*	0.361 (11)
C21	−0.0031 (5)	0.40938 (15)	0.7913 (6)	0.0136 (11)	
S1B	−0.20509 (16)	0.66393 (4)	0.60428 (19)	0.0275 (4)	
Cu1B	−0.26535 (6)	0.72732 (2)	0.69714 (7)	0.01687 (16)	
P1B	−0.44267 (14)	0.74965 (4)	0.50908 (15)	0.0135 (3)	
C22	−0.0370 (5)	0.40178 (16)	0.9099 (6)	0.0145 (11)	
H22	−0.0294	0.4238	0.9746	0.017*	
C23	−0.0817 (6)	0.36252 (17)	0.9355 (6)	0.0175 (12)	
H23	−0.1053	0.3578	1.0168	0.021*	
C24	−0.0921 (6)	0.33034 (17)	0.8432 (7)	0.0207 (13)	
H24	−0.1208	0.3033	0.8615	0.025*	
C25	−0.0601 (6)	0.33770 (17)	0.7224 (7)	0.0211 (13)	
H25	−0.0686	0.3156	0.6578	0.025*	
C26	−0.0162 (6)	0.37685 (17)	0.6963 (7)	0.0198 (12)	
H26	0.0050	0.3817	0.6137	0.024*	
C27	0.1234 (5)	0.45820 (17)	0.6242 (6)	0.0169 (11)	
H27A	0.0484	0.4449	0.5451	0.020*	
H27B	0.1372	0.4870	0.5952	0.020*	
C27B	−0.3761 (5)	0.75374 (17)	0.3619 (6)	0.0164 (11)	
H27C	−0.3580	0.7251	0.3351	0.020*	
H27D	−0.4529	0.7659	0.2817	0.020*	
C26B	−0.5248 (6)	0.83380 (17)	0.4309 (6)	0.0167 (11)	
H26B	−0.5086	0.8287	0.3460	0.020*	
C25B	−0.5646 (6)	0.87332 (18)	0.4609 (8)	0.0246 (14)	
H25B	−0.5733	0.8954	0.3963	0.029*	
C24B	−0.5916 (6)	0.88113 (17)	0.5808 (6)	0.0171 (12)	
H24B	−0.6201	0.9083	0.5984	0.021*	
C23B	−0.5771 (5)	0.84925 (17)	0.6773 (6)	0.0175 (12)	
H23B	−0.5951	0.8545	0.7613	0.021*	
C22B	−0.5362 (5)	0.80970 (16)	0.6496 (6)	0.0167 (12)	
H22B	−0.5268	0.7878	0.7151	0.020*	
C21B	−0.5089 (5)	0.80166 (16)	0.5285 (6)	0.0127 (11)	
C16B	−0.5997 (6)	0.67626 (18)	0.4434 (7)	0.0255 (13)	
H16B	−0.5107	0.6630	0.4892	0.031*	
C15B	−0.7222 (6)	0.65191 (19)	0.3874 (7)	0.0319 (16)	
H15B	−0.7169	0.6223	0.3959	0.038*	
C14B	−0.8517 (6)	0.67135 (19)	0.3194 (7)	0.0241 (13)	
H14B	−0.9355	0.6550	0.2804	0.029*	
C13B	−0.8595 (6)	0.7137 (2)	0.3081 (7)	0.0240 (13)	
H13B	−0.9487	0.7268	0.2612	0.029*	
C12B	−0.7379 (6)	0.73816 (17)	0.3646 (6)	0.0190 (12)	
H12B	−0.7445	0.7678	0.3566	0.023*	
C11B	−0.6055 (5)	0.71926 (17)	0.4332 (6)	0.0152 (11)	
C28	0.2630 (6)	0.43390 (17)	0.6502 (6)	0.0166 (11)	
H28A	0.2744	0.4288	0.5590	0.020*	
H28B	0.2532	0.4063	0.6899	0.020*	
C28B	−0.2398 (6)	0.77977 (17)	0.3860 (6)	0.0197 (12)	
H28C	−0.2540	0.8076	0.4213	0.024*	
H28D	−0.2269	0.7840	0.2950	0.024*	
C29	0.4005 (5)	0.45453 (16)	0.7469 (6)	0.0167 (11)	
H29A	0.4018	0.4842	0.7198	0.020*	
H29B	0.4838	0.4405	0.7342	0.020*	
C29B	−0.1007 (5)	0.76057 (17)	0.4887 (6)	0.0169 (11)	
H29C	−0.0180	0.7745	0.4747	0.020*	
H29D	−0.0970	0.7306	0.4659	0.020*	
C31	0.4403 (5)	0.39548 (16)	0.9623 (6)	0.0126 (10)	
C32	0.3305 (6)	0.37296 (17)	0.9855 (6)	0.0194 (12)	
H32	0.2511	0.3874	0.9951	0.023*	
C33	0.3366 (6)	0.32970 (17)	0.9945 (6)	0.0202 (12)	
H33	0.2609	0.3147	1.0096	0.024*	
C34	0.4504 (6)	0.30837 (17)	0.9820 (6)	0.0181 (12)	
H34	0.4535	0.2787	0.9875	0.022*	
C35	0.5616 (6)	0.33050 (18)	0.9610 (7)	0.0209 (12)	
H35	0.6416	0.3158	0.9540	0.025*	
C36	0.5571 (6)	0.37386 (17)	0.9502 (6)	0.0163 (12)	
H36	0.6329	0.3887	0.9346	0.020*	
C41	0.5921 (5)	0.47370 (16)	1.0282 (6)	0.0176 (12)	
C42	0.6914 (6)	0.4860 (2)	0.9675 (7)	0.0271 (14)	
H42	0.6717	0.4819	0.8706	0.032*	
C43	0.8217 (7)	0.5045 (2)	1.0501 (8)	0.0342 (16)	
H43	0.8899	0.5128	1.0091	0.041*	
C44	0.8492 (6)	0.5106 (2)	1.1905 (8)	0.0331 (17)	
H44	0.9364	0.5232	1.2462	0.040*	
C45	0.7526 (6)	0.4987 (2)	1.2494 (7)	0.0292 (15)	
H45	0.7722	0.5030	1.3462	0.035*	
C46	0.6250 (6)	0.48018 (18)	1.1690 (7)	0.0248 (13)	
H46	0.5588	0.4718	1.2122	0.030*	
C4B	−0.2630 (5)	0.70142 (16)	0.9887 (6)	0.0148 (11)	
C31B	−0.0601 (5)	0.82157 (16)	0.6981 (6)	0.0152 (11)	
C32B	−0.1698 (5)	0.84492 (17)	0.7197 (6)	0.0147 (11)	
H32B	−0.2503	0.8310	0.7287	0.018*	
C33B	−0.1614 (6)	0.88834 (18)	0.7280 (6)	0.0200 (12)	
H33B	−0.2378	0.9041	0.7385	0.024*	
C34B	−0.0405 (6)	0.90849 (18)	0.7207 (7)	0.0211 (13)	
H34B	−0.0338	0.9381	0.7283	0.025*	
C35B	0.0707 (6)	0.88569 (17)	0.7025 (7)	0.0217 (12)	
H35B	0.1532	0.8996	0.6980	0.026*	
C36B	0.0600 (6)	0.84242 (18)	0.6910 (7)	0.0189 (13)	
H36B	0.1357	0.8268	0.6782	0.023*	
C41B	0.0947 (5)	0.74421 (15)	0.7681 (6)	0.0160 (11)	
C42B	0.1932 (6)	0.73248 (18)	0.7065 (7)	0.0242 (13)	
H42B	0.1716	0.7358	0.6090	0.029*	
C43B	0.3243 (6)	0.71581 (18)	0.7887 (7)	0.0240 (13)	
H43B	0.3919	0.7079	0.7465	0.029*	
C44B	0.3570 (6)	0.71065 (18)	0.9294 (7)	0.0224 (13)	
H44B	0.4460	0.6989	0.9843	0.027*	
C45B	0.2588 (6)	0.7227 (2)	0.9907 (7)	0.0303 (15)	
H45B	0.2820	0.7200	1.0886	0.036*	
C46B	0.1269 (6)	0.73879 (18)	0.9107 (7)	0.0232 (13)	
H46B	0.0589	0.7460	0.9531	0.028*	

### Atomic displacement parameters (Å^2^)

**Table:**

	*U*^11^	*U*^22^	*U*^33^	*U*^12^	*U*^13^	*U*^23^
Cu1	0.0086 (3)	0.0155 (3)	0.0172 (4)	−0.0008 (2)	0.0019 (2)	−0.0046 (3)
S1	0.0134 (6)	0.0137 (6)	0.0220 (8)	0.0002 (4)	0.0025 (5)	0.0010 (5)
S2	0.0205 (7)	0.0356 (8)	0.0173 (8)	−0.0069 (6)	0.0055 (6)	−0.0054 (6)
P1	0.0081 (6)	0.0125 (6)	0.0176 (8)	−0.0012 (4)	−0.0001 (5)	−0.0005 (5)
P2	0.0087 (6)	0.0133 (6)	0.0149 (7)	0.0016 (4)	0.0022 (5)	−0.0005 (5)
N1	0.020 (2)	0.022 (2)	0.030 (3)	−0.0079 (18)	0.008 (2)	−0.003 (2)
N2	0.021 (2)	0.021 (2)	0.029 (3)	−0.0091 (18)	0.006 (2)	0.002 (2)
N3	0.012 (2)	0.023 (2)	0.025 (3)	−0.0028 (16)	0.007 (2)	−0.005 (2)
C1	0.011 (2)	0.012 (2)	0.027 (4)	0.0018 (17)	0.005 (2)	0.000 (2)
C2	0.051 (5)	0.037 (4)	0.046 (5)	−0.022 (3)	0.025 (4)	−0.008 (3)
S2B	0.0154 (6)	0.0307 (7)	0.0172 (8)	−0.0035 (5)	0.0024 (5)	0.0025 (6)
C1B	0.017 (3)	0.008 (3)	0.015 (5)	0.0004 (19)	0.004 (4)	−0.002 (4)
N2B	0.018 (4)	0.022 (5)	0.015 (5)	0.007 (3)	0.004 (3)	0.000 (3)
C3B	0.040 (6)	0.018 (7)	0.022 (5)	0.010 (5)	−0.010 (4)	−0.004 (4)
N1B	0.021 (4)	0.025 (6)	0.022 (6)	0.011 (4)	0.007 (4)	0.002 (4)
C2B	0.047 (7)	0.055 (8)	0.026 (6)	0.025 (5)	0.015 (5)	0.003 (4)
C1C	0.017 (3)	0.008 (3)	0.015 (5)	0.0004 (19)	0.004 (4)	−0.002 (4)
N2C	0.018 (4)	0.022 (5)	0.015 (5)	0.007 (3)	0.004 (3)	0.000 (3)
C3C	0.040 (6)	0.018 (7)	0.022 (5)	0.010 (5)	−0.010 (4)	−0.004 (4)
N1C	0.021 (4)	0.025 (6)	0.022 (6)	0.011 (4)	0.007 (4)	0.002 (4)
C2C	0.047 (7)	0.055 (8)	0.026 (6)	0.025 (5)	0.015 (5)	0.003 (4)
P2B	0.0086 (6)	0.0156 (6)	0.0172 (8)	−0.0001 (4)	0.0035 (5)	0.0021 (5)
C3	0.034 (4)	0.030 (3)	0.030 (4)	−0.014 (3)	−0.001 (3)	0.001 (3)
N3B	0.014 (2)	0.024 (2)	0.034 (3)	−0.0022 (17)	0.005 (2)	0.005 (2)
C4	0.008 (2)	0.018 (2)	0.020 (3)	−0.0029 (17)	0.003 (2)	0.001 (2)
C11	0.011 (2)	0.014 (2)	0.022 (4)	−0.0019 (19)	−0.001 (2)	0.001 (2)
C12	0.014 (2)	0.014 (4)	0.017 (7)	0.001 (3)	0.000 (3)	0.003 (4)
C13	0.017 (4)	0.020 (6)	0.008 (5)	0.000 (4)	0.000 (3)	0.007 (5)
C14	0.013 (2)	0.019 (4)	0.042 (9)	0.002 (2)	0.006 (3)	0.004 (4)
C15	0.015 (3)	0.014 (3)	0.040 (7)	0.000 (2)	0.009 (4)	−0.001 (4)
C16	0.012 (3)	0.018 (3)	0.037 (8)	−0.0030 (19)	0.010 (4)	0.001 (4)
C11C	0.011 (2)	0.014 (2)	0.022 (4)	−0.0019 (19)	−0.001 (2)	0.001 (2)
C12C	0.014 (2)	0.014 (4)	0.017 (7)	0.001 (3)	0.000 (3)	0.003 (4)
C13C	0.014 (2)	0.014 (4)	0.017 (7)	0.001 (3)	0.000 (3)	0.003 (4)
C14C	0.013 (2)	0.019 (4)	0.042 (9)	0.002 (2)	0.006 (3)	0.004 (4)
C15C	0.015 (3)	0.014 (3)	0.040 (7)	0.000 (2)	0.009 (4)	−0.001 (4)
C16C	0.012 (3)	0.018 (3)	0.037 (8)	−0.0030 (19)	0.010 (4)	0.001 (4)
C21	0.010 (2)	0.013 (2)	0.016 (3)	−0.0005 (17)	0.002 (2)	−0.002 (2)
S1B	0.0211 (7)	0.0174 (7)	0.0390 (10)	0.0079 (5)	0.0039 (7)	0.0061 (6)
Cu1B	0.0091 (3)	0.0173 (3)	0.0221 (4)	0.0009 (2)	0.0026 (3)	0.0066 (3)
P1B	0.0091 (6)	0.0126 (6)	0.0172 (8)	0.0006 (4)	0.0024 (5)	0.0012 (5)
C22	0.013 (2)	0.014 (2)	0.011 (3)	0.0011 (17)	−0.003 (2)	−0.003 (2)
C23	0.012 (2)	0.023 (3)	0.016 (3)	−0.0002 (18)	0.003 (2)	0.005 (2)
C24	0.016 (3)	0.014 (2)	0.029 (4)	−0.0015 (19)	0.004 (2)	−0.001 (2)
C25	0.024 (3)	0.015 (3)	0.023 (4)	−0.004 (2)	0.007 (3)	−0.006 (2)
C26	0.019 (3)	0.019 (3)	0.019 (3)	−0.004 (2)	0.004 (2)	−0.003 (2)
C27	0.015 (2)	0.019 (2)	0.013 (3)	−0.0005 (19)	0.000 (2)	0.003 (2)
C27B	0.012 (2)	0.023 (3)	0.016 (3)	−0.0004 (18)	0.007 (2)	−0.003 (2)
C26B	0.019 (3)	0.022 (3)	0.010 (3)	0.0022 (19)	0.006 (2)	0.001 (2)
C25B	0.019 (3)	0.017 (3)	0.039 (4)	0.005 (2)	0.012 (3)	0.011 (3)
C24B	0.015 (3)	0.015 (2)	0.018 (3)	0.0049 (19)	0.002 (2)	0.001 (2)
C23B	0.011 (2)	0.023 (3)	0.015 (3)	−0.0001 (19)	0.001 (2)	0.000 (2)
C22B	0.011 (2)	0.013 (2)	0.020 (3)	−0.0019 (17)	−0.002 (2)	0.004 (2)
C21B	0.006 (2)	0.014 (2)	0.014 (3)	−0.0003 (16)	−0.0027 (19)	−0.002 (2)
C16B	0.016 (3)	0.021 (3)	0.034 (4)	−0.002 (2)	0.001 (2)	0.000 (2)
C15B	0.022 (3)	0.021 (3)	0.043 (4)	−0.006 (2)	−0.001 (3)	−0.002 (3)
C14B	0.016 (3)	0.033 (3)	0.025 (3)	−0.010 (2)	0.009 (2)	−0.009 (3)
C13B	0.006 (2)	0.041 (3)	0.024 (3)	−0.002 (2)	0.003 (2)	−0.006 (3)
C12B	0.016 (3)	0.020 (3)	0.018 (3)	−0.0010 (18)	0.001 (2)	−0.002 (2)
C11B	0.009 (2)	0.024 (3)	0.012 (3)	−0.0011 (18)	0.0027 (19)	−0.002 (2)
C28	0.017 (3)	0.022 (3)	0.010 (3)	0.0007 (19)	0.004 (2)	−0.001 (2)
C28B	0.016 (3)	0.022 (3)	0.018 (3)	0.0011 (19)	0.001 (2)	0.006 (2)
C29	0.013 (2)	0.016 (2)	0.020 (3)	0.0001 (18)	0.004 (2)	−0.001 (2)
C29B	0.012 (2)	0.023 (3)	0.019 (3)	−0.0038 (19)	0.009 (2)	0.001 (2)
C31	0.014 (2)	0.018 (2)	0.006 (3)	0.0031 (18)	0.0025 (19)	0.000 (2)
C32	0.011 (2)	0.019 (3)	0.023 (3)	0.0024 (18)	0.000 (2)	0.003 (2)
C33	0.015 (3)	0.023 (3)	0.016 (3)	−0.0052 (19)	−0.003 (2)	0.001 (2)
C34	0.028 (3)	0.017 (3)	0.006 (3)	0.001 (2)	0.002 (2)	0.000 (2)
C35	0.022 (3)	0.022 (3)	0.016 (3)	0.008 (2)	0.004 (2)	−0.002 (2)
C36	0.020 (3)	0.020 (3)	0.010 (3)	0.002 (2)	0.006 (2)	−0.001 (2)
C41	0.010 (2)	0.016 (2)	0.025 (3)	0.0033 (17)	0.002 (2)	−0.003 (2)
C42	0.019 (3)	0.045 (4)	0.021 (3)	−0.007 (3)	0.012 (2)	−0.013 (3)
C43	0.016 (3)	0.046 (4)	0.044 (5)	−0.010 (3)	0.015 (3)	−0.012 (3)
C44	0.009 (3)	0.033 (3)	0.050 (5)	−0.001 (2)	0.000 (3)	−0.016 (3)
C45	0.019 (3)	0.037 (3)	0.023 (4)	0.001 (2)	−0.003 (2)	−0.010 (3)
C46	0.018 (3)	0.027 (3)	0.029 (4)	0.000 (2)	0.007 (2)	−0.001 (3)
C4B	0.011 (2)	0.018 (2)	0.017 (3)	−0.0019 (18)	0.007 (2)	−0.005 (2)
C31B	0.014 (2)	0.014 (2)	0.014 (3)	0.0010 (18)	0.000 (2)	0.001 (2)
C32B	0.010 (2)	0.027 (3)	0.008 (3)	0.0006 (19)	0.004 (2)	−0.002 (2)
C33B	0.019 (3)	0.027 (3)	0.013 (3)	0.003 (2)	0.004 (2)	0.000 (2)
C34B	0.020 (3)	0.016 (3)	0.023 (3)	0.000 (2)	0.000 (2)	0.004 (2)
C35B	0.022 (3)	0.020 (3)	0.022 (3)	−0.004 (2)	0.006 (2)	0.002 (2)
C36B	0.011 (3)	0.019 (3)	0.026 (4)	0.0009 (19)	0.007 (2)	0.001 (2)
C41B	0.013 (2)	0.010 (2)	0.022 (3)	−0.0036 (17)	0.002 (2)	0.002 (2)
C42B	0.015 (3)	0.025 (3)	0.029 (4)	0.002 (2)	0.003 (2)	0.004 (3)
C43B	0.014 (3)	0.025 (3)	0.039 (4)	0.003 (2)	0.018 (3)	0.005 (3)
C44B	0.019 (3)	0.019 (3)	0.024 (4)	0.000 (2)	0.001 (2)	0.003 (2)
C45B	0.016 (3)	0.038 (3)	0.030 (4)	0.004 (2)	−0.001 (2)	−0.001 (3)
C46B	0.012 (3)	0.030 (3)	0.023 (3)	0.003 (2)	0.001 (2)	−0.003 (2)

### Geometric parameters (Å, º)

**Table:**

Cu1—N3	1.957 (5)	C23—C24	1.375 (8)
Cu1—P1	2.2348 (15)	C23—H23	0.9500
Cu1—P2	2.2683 (14)	C24—C25	1.395 (10)
Cu1—S1	2.4292 (15)	C24—H24	0.9500
S1—C1	1.724 (6)	C25—C26	1.382 (8)
S2—C4	1.634 (6)	C25—H25	0.9500
P1—C21	1.817 (5)	C26—H26	0.9500
P1—C11C	1.82 (3)	C27—C28	1.536 (7)
P1—C27	1.838 (6)	C27—H27A	0.9900
P1—C11	1.842 (17)	C27—H27B	0.9900
P2—C41	1.816 (6)	C27B—C28B	1.540 (7)
P2—C31	1.828 (5)	C27B—H27C	0.9900
P2—C29	1.852 (6)	C27B—H27D	0.9900
N1—C1	1.336 (7)	C26B—C25B	1.390 (8)
N1—C2	1.447 (10)	C26B—C21B	1.402 (8)
N1—H1	0.8800	C26B—H26B	0.9500
N2—C1	1.312 (8)	C25B—C24B	1.364 (10)
N2—C3	1.461 (8)	C25B—H25B	0.9500
N2—H2	0.8800	C24B—C23B	1.391 (8)
N3—C4	1.149 (8)	C24B—H24B	0.9500
C2—H2C	0.9800	C23B—C22B	1.387 (8)
C2—H2D	0.9800	C23B—H23B	0.9500
C2—H2E	0.9800	C22B—C21B	1.379 (9)
S2B—C4B	1.654 (6)	C22B—H22B	0.9500
C1B—N2B	1.322 (12)	C16B—C11B	1.378 (8)
C1B—N1B	1.332 (11)	C16B—C15B	1.395 (8)
C1B—S1B	1.675 (14)	C16B—H16B	0.9500
N2B—C3B	1.467 (11)	C15B—C14B	1.387 (9)
N2B—H2L	0.8800	C15B—H15B	0.9500
C3B—H3BA	0.9800	C14B—C13B	1.360 (9)
C3B—H3BB	0.9800	C14B—H14B	0.9500
C3B—H3BC	0.9800	C13B—C12B	1.391 (7)
N1B—C2B	1.438 (14)	C13B—H13B	0.9500
N1B—H1B	0.8800	C12B—C11B	1.402 (7)
C2B—H2F	0.9800	C12B—H12B	0.9500
C2B—H2G	0.9800	C28—C29	1.537 (7)
C2B—H2H	0.9800	C28—H28A	0.9900
C1C—N1C	1.320 (18)	C28—H28B	0.9900
C1C—N2C	1.335 (19)	C28B—C29B	1.551 (8)
C1C—S1B	1.82 (3)	C28B—H28C	0.9900
N2C—C3C	1.466 (18)	C28B—H28D	0.9900
N2C—H2M	0.8800	C29—H29A	0.9900
C3C—H3BD	0.9800	C29—H29B	0.9900
C3C—H3BE	0.9800	C29B—H29C	0.9900
C3C—H3BF	0.9800	C29B—H29D	0.9900
N1C—C2C	1.43 (2)	C31—C36	1.396 (8)
N1C—H1C	0.8800	C31—C32	1.397 (8)
C2C—H2I	0.9800	C32—C33	1.386 (8)
C2C—H2J	0.9800	C32—H32	0.9500
C2C—H2K	0.9800	C33—C34	1.367 (9)
P2B—C29B	1.824 (6)	C33—H33	0.9500
P2B—C31B	1.827 (5)	C34—C35	1.392 (9)
P2B—C41B	1.836 (5)	C34—H34	0.9500
P2B—Cu1B	2.2688 (15)	C35—C36	1.390 (8)
C3—H3A	0.9800	C35—H35	0.9500
C3—H3B	0.9800	C36—H36	0.9500
C3—H3C	0.9800	C41—C46	1.377 (9)
N3B—C4B	1.152 (8)	C41—C42	1.393 (9)
N3B—Cu1B	1.954 (6)	C42—C43	1.415 (9)
C11—C16	1.384 (14)	C42—H42	0.9500
C11—C12	1.387 (12)	C43—C44	1.378 (11)
C12—C13	1.412 (11)	C43—H43	0.9500
C12—H12	0.9500	C44—C45	1.354 (10)
C13—C14	1.358 (13)	C44—H44	0.9500
C13—H13	0.9500	C45—C46	1.388 (8)
C14—C15	1.387 (13)	C45—H45	0.9500
C14—H14	0.9500	C46—H46	0.9500
C15—C16	1.406 (11)	C31B—C36B	1.394 (8)
C15—H15	0.9500	C31B—C32B	1.403 (7)
C16—H16	0.9500	C32B—C33B	1.391 (8)
C11C—C16C	1.379 (19)	C32B—H32B	0.9500
C11C—C12C	1.389 (18)	C33B—C34B	1.391 (9)
C12C—C13C	1.394 (18)	C33B—H33B	0.9500
C12C—H12C	0.9500	C34B—C35B	1.392 (9)
C13C—C14C	1.346 (19)	C34B—H34B	0.9500
C13C—H13C	0.9500	C35B—C36B	1.390 (8)
C14C—C15C	1.40 (2)	C35B—H35B	0.9500
C14C—H14C	0.9500	C36B—H36B	0.9500
C15C—C16C	1.411 (17)	C41B—C42B	1.388 (9)
C15C—H15C	0.9500	C41B—C46B	1.391 (9)
C16C—H16C	0.9500	C42B—C43B	1.395 (8)
C21—C22	1.387 (9)	C42B—H42B	0.9500
C21—C26	1.398 (8)	C43B—C44B	1.370 (10)
S1B—Cu1B	2.3997 (17)	C43B—H43B	0.9500
Cu1B—P1B	2.2363 (15)	C44B—C45B	1.386 (10)
P1B—C11B	1.822 (5)	C44B—H44B	0.9500
P1B—C21B	1.825 (5)	C45B—C46B	1.388 (8)
P1B—C27B	1.845 (6)	C45B—H45B	0.9500
C22—C23	1.387 (7)	C46B—H46B	0.9500
C22—H22	0.9500		
			
N3—Cu1—P1	127.47 (14)	C23—C24—H24	120.2
N3—Cu1—P2	120.47 (14)	C25—C24—H24	120.2
P1—Cu1—P2	98.95 (6)	C26—C25—C24	120.5 (6)
N3—Cu1—S1	108.54 (15)	C26—C25—H25	119.8
P1—Cu1—S1	101.73 (5)	C24—C25—H25	119.8
P2—Cu1—S1	93.27 (5)	C25—C26—C21	120.0 (6)
C1—S1—Cu1	109.4 (2)	C25—C26—H26	120.0
C21—P1—C11C	100.8 (10)	C21—C26—H26	120.0
C21—P1—C27	105.0 (3)	C28—C27—P1	116.8 (4)
C11C—P1—C27	100.9 (16)	C28—C27—H27A	108.1
C21—P1—C11	101.7 (6)	P1—C27—H27A	108.1
C27—P1—C11	104.0 (9)	C28—C27—H27B	108.1
C21—P1—Cu1	116.34 (19)	P1—C27—H27B	108.1
C11C—P1—Cu1	123.3 (10)	H27A—C27—H27B	107.3
C27—P1—Cu1	108.22 (17)	C28B—C27B—P1B	116.9 (4)
C11—P1—Cu1	120.0 (6)	C28B—C27B—H27C	108.1
C41—P2—C31	104.5 (2)	P1B—C27B—H27C	108.1
C41—P2—C29	105.5 (3)	C28B—C27B—H27D	108.1
C31—P2—C29	100.2 (2)	P1B—C27B—H27D	108.1
C41—P2—Cu1	114.79 (18)	H27C—C27B—H27D	107.3
C31—P2—Cu1	123.29 (18)	C25B—C26B—C21B	118.8 (6)
C29—P2—Cu1	106.41 (17)	C25B—C26B—H26B	120.6
C1—N1—C2	123.9 (5)	C21B—C26B—H26B	120.6
C1—N1—H1	118.0	C24B—C25B—C26B	121.5 (6)
C2—N1—H1	118.0	C24B—C25B—H25B	119.2
C1—N2—C3	124.6 (5)	C26B—C25B—H25B	119.2
C1—N2—H2	117.7	C25B—C24B—C23B	119.9 (5)
C3—N2—H2	117.7	C25B—C24B—H24B	120.1
C4—N3—Cu1	171.7 (4)	C23B—C24B—H24B	120.1
N2—C1—N1	119.7 (5)	C22B—C23B—C24B	119.3 (6)
N2—C1—S1	120.2 (4)	C22B—C23B—H23B	120.3
N1—C1—S1	120.1 (5)	C24B—C23B—H23B	120.3
N1—C2—H2C	109.5	C21B—C22B—C23B	121.0 (5)
N1—C2—H2D	109.5	C21B—C22B—H22B	119.5
H2C—C2—H2D	109.5	C23B—C22B—H22B	119.5
N1—C2—H2E	109.5	C22B—C21B—C26B	119.5 (5)
H2C—C2—H2E	109.5	C22B—C21B—P1B	117.2 (4)
H2D—C2—H2E	109.5	C26B—C21B—P1B	123.2 (5)
N2B—C1B—N1B	118.9 (11)	C11B—C16B—C15B	121.1 (5)
N2B—C1B—S1B	117.3 (9)	C11B—C16B—H16B	119.4
N1B—C1B—S1B	123.8 (9)	C15B—C16B—H16B	119.4
C1B—N2B—C3B	126.0 (11)	C14B—C15B—C16B	119.3 (5)
C1B—N2B—H2L	117.0	C14B—C15B—H15B	120.3
C3B—N2B—H2L	117.0	C16B—C15B—H15B	120.3
N2B—C3B—H3BA	109.5	C13B—C14B—C15B	120.4 (5)
N2B—C3B—H3BB	109.5	C13B—C14B—H14B	119.8
H3BA—C3B—H3BB	109.5	C15B—C14B—H14B	119.8
N2B—C3B—H3BC	109.5	C14B—C13B—C12B	120.5 (5)
H3BA—C3B—H3BC	109.5	C14B—C13B—H13B	119.7
H3BB—C3B—H3BC	109.5	C12B—C13B—H13B	119.7
C1B—N1B—C2B	123.1 (11)	C13B—C12B—C11B	120.3 (5)
C1B—N1B—H1B	118.4	C13B—C12B—H12B	119.9
C2B—N1B—H1B	118.4	C11B—C12B—H12B	119.9
N1B—C2B—H2F	109.5	C16B—C11B—C12B	118.4 (5)
N1B—C2B—H2G	109.5	C16B—C11B—P1B	119.4 (4)
H2F—C2B—H2G	109.5	C12B—C11B—P1B	122.2 (4)
N1B—C2B—H2H	109.5	C27—C28—C29	116.8 (5)
H2F—C2B—H2H	109.5	C27—C28—H28A	108.1
H2G—C2B—H2H	109.5	C29—C28—H28A	108.1
N1C—C1C—N2C	119 (2)	C27—C28—H28B	108.1
N1C—C1C—S1B	115.5 (16)	C29—C28—H28B	108.1
N2C—C1C—S1B	125.0 (17)	H28A—C28—H28B	107.3
C1C—N2C—C3C	122 (2)	C27B—C28B—C29B	115.5 (5)
C1C—N2C—H2M	119.2	C27B—C28B—H28C	108.4
C3C—N2C—H2M	119.2	C29B—C28B—H28C	108.4
N2C—C3C—H3BD	109.5	C27B—C28B—H28D	108.4
N2C—C3C—H3BE	109.5	C29B—C28B—H28D	108.4
H3BD—C3C—H3BE	109.5	H28C—C28B—H28D	107.5
N2C—C3C—H3BF	109.5	C28—C29—P2	112.9 (4)
H3BD—C3C—H3BF	109.5	C28—C29—H29A	109.0
H3BE—C3C—H3BF	109.5	P2—C29—H29A	109.0
C1C—N1C—C2C	125 (2)	C28—C29—H29B	109.0
C1C—N1C—H1C	117.4	P2—C29—H29B	109.0
C2C—N1C—H1C	117.4	H29A—C29—H29B	107.8
N1C—C2C—H2I	109.5	C28B—C29B—P2B	114.3 (4)
N1C—C2C—H2J	109.5	C28B—C29B—H29C	108.7
H2I—C2C—H2J	109.5	P2B—C29B—H29C	108.7
N1C—C2C—H2K	109.5	C28B—C29B—H29D	108.7
H2I—C2C—H2K	109.5	P2B—C29B—H29D	108.7
H2J—C2C—H2K	109.5	H29C—C29B—H29D	107.6
C29B—P2B—C31B	101.1 (3)	C36—C31—C32	119.1 (5)
C29B—P2B—C41B	104.8 (3)	C36—C31—P2	122.2 (4)
C31B—P2B—C41B	103.3 (2)	C32—C31—P2	118.5 (4)
C29B—P2B—Cu1B	106.01 (17)	C33—C32—C31	120.4 (5)
C31B—P2B—Cu1B	125.14 (19)	C33—C32—H32	119.8
C41B—P2B—Cu1B	114.14 (17)	C31—C32—H32	119.8
N2—C3—H3A	109.5	C34—C33—C32	120.7 (6)
N2—C3—H3B	109.5	C34—C33—H33	119.7
H3A—C3—H3B	109.5	C32—C33—H33	119.7
N2—C3—H3C	109.5	C33—C34—C35	119.4 (5)
H3A—C3—H3C	109.5	C33—C34—H34	120.3
H3B—C3—H3C	109.5	C35—C34—H34	120.3
C4B—N3B—Cu1B	165.8 (5)	C36—C35—C34	120.9 (5)
N3—C4—S2	178.5 (5)	C36—C35—H35	119.6
C16—C11—C12	120.1 (12)	C34—C35—H35	119.6
C16—C11—P1	118.2 (11)	C35—C36—C31	119.5 (6)
C12—C11—P1	121.6 (10)	C35—C36—H36	120.3
C11—C12—C13	119.5 (11)	C31—C36—H36	120.3
C11—C12—H12	120.2	C46—C41—C42	118.1 (5)
C13—C12—H12	120.2	C46—C41—P2	117.3 (5)
C14—C13—C12	120.5 (11)	C42—C41—P2	124.5 (5)
C14—C13—H13	119.8	C41—C42—C43	120.0 (6)
C12—C13—H13	119.8	C41—C42—H42	120.0
C13—C14—C15	120.0 (11)	C43—C42—H42	120.0
C13—C14—H14	120.0	C44—C43—C42	119.6 (6)
C15—C14—H14	120.0	C44—C43—H43	120.2
C14—C15—C16	120.2 (10)	C42—C43—H43	120.2
C14—C15—H15	119.9	C45—C44—C43	120.3 (6)
C16—C15—H15	119.9	C45—C44—H44	119.8
C11—C16—C15	119.5 (10)	C43—C44—H44	119.8
C11—C16—H16	120.3	C44—C45—C46	120.3 (7)
C15—C16—H16	120.3	C44—C45—H45	119.9
C16C—C11C—C12C	120 (2)	C46—C45—H45	119.9
C16C—C11C—P1	119.5 (19)	C41—C46—C45	121.7 (6)
C12C—C11C—P1	121.0 (19)	C41—C46—H46	119.2
C11C—C12C—C13C	119.9 (19)	C45—C46—H46	119.2
C11C—C12C—H12C	120.1	N3B—C4B—S2B	178.5 (5)
C13C—C12C—H12C	120.1	C36B—C31B—C32B	118.9 (5)
C14C—C13C—C12C	120.5 (19)	C36B—C31B—P2B	122.2 (4)
C14C—C13C—H13C	119.7	C32B—C31B—P2B	118.8 (4)
C12C—C13C—H13C	119.7	C33B—C32B—C31B	120.4 (5)
C13C—C14C—C15C	121 (2)	C33B—C32B—H32B	119.8
C13C—C14C—H14C	119.4	C31B—C32B—H32B	119.8
C15C—C14C—H14C	119.4	C34B—C33B—C32B	119.6 (5)
C14C—C15C—C16C	118.1 (17)	C34B—C33B—H33B	120.2
C14C—C15C—H15C	120.9	C32B—C33B—H33B	120.2
C16C—C15C—H15C	120.9	C33B—C34B—C35B	120.6 (5)
C11C—C16C—C15C	120.3 (18)	C33B—C34B—H34B	119.7
C11C—C16C—H16C	119.8	C35B—C34B—H34B	119.7
C15C—C16C—H16C	119.8	C36B—C35B—C34B	119.4 (5)
C22—C21—C26	118.9 (5)	C36B—C35B—H35B	120.3
C22—C21—P1	116.8 (4)	C34B—C35B—H35B	120.3
C26—C21—P1	124.3 (5)	C35B—C36B—C31B	120.9 (5)
C1B—S1B—Cu1B	111.4 (5)	C35B—C36B—H36B	119.5
C1C—S1B—Cu1B	103.0 (10)	C31B—C36B—H36B	119.5
N3B—Cu1B—P1B	127.96 (15)	C42B—C41B—C46B	119.7 (5)
N3B—Cu1B—P2B	120.23 (15)	C42B—C41B—P2B	124.1 (5)
P1B—Cu1B—P2B	99.20 (6)	C46B—C41B—P2B	116.2 (4)
N3B—Cu1B—S1B	108.75 (15)	C41B—C42B—C43B	119.6 (6)
P1B—Cu1B—S1B	99.28 (6)	C41B—C42B—H42B	120.2
P2B—Cu1B—S1B	94.95 (6)	C43B—C42B—H42B	120.2
C11B—P1B—C21B	103.2 (2)	C44B—C43B—C42B	120.9 (6)
C11B—P1B—C27B	102.0 (3)	C44B—C43B—H43B	119.5
C21B—P1B—C27B	105.6 (3)	C42B—C43B—H43B	119.5
C11B—P1B—Cu1B	121.51 (19)	C43B—C44B—C45B	119.3 (6)
C21B—P1B—Cu1B	114.00 (19)	C43B—C44B—H44B	120.3
C27B—P1B—Cu1B	108.87 (18)	C45B—C44B—H44B	120.3
C23—C22—C21	120.9 (5)	C44B—C45B—C46B	120.7 (7)
C23—C22—H22	119.6	C44B—C45B—H45B	119.6
C21—C22—H22	119.6	C46B—C45B—H45B	119.6
C24—C23—C22	120.1 (6)	C45B—C46B—C41B	119.7 (6)
C24—C23—H23	119.9	C45B—C46B—H46B	120.2
C22—C23—H23	119.9	C41B—C46B—H46B	120.2
C23—C24—C25	119.6 (5)		
			
C3—N2—C1—N1	−3.0 (9)	Cu1B—P1B—C21B—C26B	129.4 (4)
C3—N2—C1—S1	177.9 (5)	C11B—C16B—C15B—C14B	0.6 (11)
C2—N1—C1—N2	178.9 (6)	C16B—C15B—C14B—C13B	−0.4 (11)
C2—N1—C1—S1	−2.0 (8)	C15B—C14B—C13B—C12B	0.0 (10)
Cu1—S1—C1—N2	−24.5 (5)	C14B—C13B—C12B—C11B	0.4 (10)
Cu1—S1—C1—N1	156.4 (4)	C15B—C16B—C11B—C12B	−0.3 (10)
N1B—C1B—N2B—C3B	3 (2)	C15B—C16B—C11B—P1B	179.6 (6)
S1B—C1B—N2B—C3B	−177.5 (10)	C13B—C12B—C11B—C16B	−0.2 (9)
N2B—C1B—N1B—C2B	172.5 (16)	C13B—C12B—C11B—P1B	179.9 (5)
S1B—C1B—N1B—C2B	−7 (3)	C21B—P1B—C11B—C16B	−159.8 (5)
N1C—C1C—N2C—C3C	3 (4)	C27B—P1B—C11B—C16B	90.8 (5)
S1B—C1C—N2C—C3C	173 (2)	Cu1B—P1B—C11B—C16B	−30.4 (6)
N2C—C1C—N1C—C2C	176 (3)	C21B—P1B—C11B—C12B	20.1 (6)
S1B—C1C—N1C—C2C	5 (4)	C27B—P1B—C11B—C12B	−89.4 (5)
C21—P1—C11—C16	139.8 (17)	Cu1B—P1B—C11B—C12B	149.4 (4)
C27—P1—C11—C16	−111.3 (18)	P1—C27—C28—C29	−70.8 (6)
Cu1—P1—C11—C16	10 (2)	P1B—C27B—C28B—C29B	69.2 (6)
C21—P1—C11—C12	−36.9 (16)	C27—C28—C29—P2	74.9 (6)
C27—P1—C11—C12	72.0 (15)	C41—P2—C29—C28	175.3 (4)
Cu1—P1—C11—C12	−166.9 (12)	C31—P2—C29—C28	67.0 (4)
C16—C11—C12—C13	0.8 (19)	Cu1—P2—C29—C28	−62.3 (4)
P1—C11—C12—C13	177.4 (17)	C27B—C28B—C29B—P2B	−75.8 (6)
C11—C12—C13—C14	3.6 (16)	C31B—P2B—C29B—C28B	−68.3 (4)
C12—C13—C14—C15	−6 (3)	C41B—P2B—C29B—C28B	−175.4 (4)
C13—C14—C15—C16	4 (3)	Cu1B—P2B—C29B—C28B	63.5 (4)
C12—C11—C16—C15	−3 (2)	C41—P2—C31—C36	−45.9 (5)
P1—C11—C16—C15	−179.3 (12)	C29—P2—C31—C36	63.2 (5)
C14—C15—C16—C11	0 (2)	Cu1—P2—C31—C36	−179.3 (4)
C21—P1—C11C—C16C	161 (3)	C41—P2—C31—C32	139.8 (5)
C27—P1—C11C—C16C	−91 (3)	C29—P2—C31—C32	−111.1 (5)
Cu1—P1—C11C—C16C	29 (4)	Cu1—P2—C31—C32	6.4 (5)
C21—P1—C11C—C12C	−17 (3)	C36—C31—C32—C33	−0.8 (9)
C27—P1—C11C—C12C	91 (3)	P2—C31—C32—C33	173.6 (5)
Cu1—P1—C11C—C12C	−149 (2)	C31—C32—C33—C34	0.5 (9)
C16C—C11C—C12C—C13C	1 (3)	C32—C33—C34—C35	0.5 (9)
P1—C11C—C12C—C13C	179 (4)	C33—C34—C35—C36	−1.2 (9)
C11C—C12C—C13C—C14C	−1 (3)	C34—C35—C36—C31	0.8 (9)
C12C—C13C—C14C—C15C	4 (5)	C32—C31—C36—C35	0.2 (9)
C13C—C14C—C15C—C16C	−7 (5)	P2—C31—C36—C35	−174.1 (5)
C12C—C11C—C16C—C15C	−4 (4)	C31—P2—C41—C46	−82.7 (5)
P1—C11C—C16C—C15C	178 (2)	C29—P2—C41—C46	172.2 (4)
C14C—C15C—C16C—C11C	7 (4)	Cu1—P2—C41—C46	55.4 (5)
C11C—P1—C21—C22	−85.8 (16)	C31—P2—C41—C42	100.3 (5)
C27—P1—C21—C22	169.7 (4)	C29—P2—C41—C42	−4.8 (6)
C11—P1—C21—C22	−82.2 (9)	Cu1—P2—C41—C42	−121.7 (5)
Cu1—P1—C21—C22	50.1 (4)	C46—C41—C42—C43	−0.1 (9)
C11C—P1—C21—C26	94.4 (16)	P2—C41—C42—C43	177.0 (5)
C27—P1—C21—C26	−10.1 (5)	C41—C42—C43—C44	−0.3 (10)
C11—P1—C21—C26	98.1 (10)	C42—C43—C44—C45	0.3 (10)
Cu1—P1—C21—C26	−129.7 (4)	C43—C44—C45—C46	0.1 (10)
N2B—C1B—S1B—Cu1B	28.9 (17)	C42—C41—C46—C45	0.5 (9)
N1B—C1B—S1B—Cu1B	−151.7 (15)	P2—C41—C46—C45	−176.7 (5)
N1C—C1C—S1B—Cu1B	−142 (3)	C44—C45—C46—C41	−0.5 (9)
N2C—C1C—S1B—Cu1B	48 (3)	C29B—P2B—C31B—C36B	−66.2 (5)
C26—C21—C22—C23	0.7 (8)	C41B—P2B—C31B—C36B	42.1 (6)
P1—C21—C22—C23	−179.1 (4)	Cu1B—P2B—C31B—C36B	175.0 (4)
C21—C22—C23—C24	0.5 (8)	C29B—P2B—C31B—C32B	110.8 (5)
C22—C23—C24—C25	−1.3 (8)	C41B—P2B—C31B—C32B	−140.9 (5)
C23—C24—C25—C26	1.0 (9)	Cu1B—P2B—C31B—C32B	−8.0 (5)
C24—C25—C26—C21	0.2 (9)	C36B—C31B—C32B—C33B	2.7 (8)
C22—C21—C26—C25	−1.1 (8)	P2B—C31B—C32B—C33B	−174.4 (4)
P1—C21—C26—C25	178.7 (4)	C31B—C32B—C33B—C34B	−2.8 (8)
C21—P1—C27—C28	−70.4 (4)	C32B—C33B—C34B—C35B	1.3 (9)
C11C—P1—C27—C28	−174.8 (9)	C33B—C34B—C35B—C36B	0.2 (10)
C11—P1—C27—C28	−176.8 (6)	C34B—C35B—C36B—C31B	−0.3 (10)
Cu1—P1—C27—C28	54.5 (4)	C32B—C31B—C36B—C35B	−1.2 (9)
C11B—P1B—C27B—C28B	177.2 (4)	P2B—C31B—C36B—C35B	175.8 (5)
C21B—P1B—C27B—C28B	69.6 (4)	C29B—P2B—C41B—C42B	6.2 (5)
Cu1B—P1B—C27B—C28B	−53.1 (4)	C31B—P2B—C41B—C42B	−99.3 (5)
C21B—C26B—C25B—C24B	−1.5 (9)	Cu1B—P2B—C41B—C42B	121.7 (4)
C26B—C25B—C24B—C23B	1.0 (9)	C29B—P2B—C41B—C46B	−171.4 (4)
C25B—C24B—C23B—C22B	−0.4 (8)	C31B—P2B—C41B—C46B	83.1 (5)
C24B—C23B—C22B—C21B	0.4 (8)	Cu1B—P2B—C41B—C46B	−55.8 (4)
C23B—C22B—C21B—C26B	−0.9 (8)	C46B—C41B—C42B—C43B	−0.7 (8)
C23B—C22B—C21B—P1B	175.4 (4)	P2B—C41B—C42B—C43B	−178.2 (4)
C25B—C26B—C21B—C22B	1.4 (8)	C41B—C42B—C43B—C44B	0.3 (9)
C25B—C26B—C21B—P1B	−174.6 (4)	C42B—C43B—C44B—C45B	−0.9 (9)
C11B—P1B—C21B—C22B	87.1 (4)	C43B—C44B—C45B—C46B	1.8 (9)
C27B—P1B—C21B—C22B	−166.2 (4)	C44B—C45B—C46B—C41B	−2.3 (9)
Cu1B—P1B—C21B—C22B	−46.7 (4)	C42B—C41B—C46B—C45B	1.7 (8)
C11B—P1B—C21B—C26B	−96.8 (5)	P2B—C41B—C46B—C45B	179.4 (5)
C27B—P1B—C21B—C26B	10.0 (5)		

### Hydrogen-bond geometry (Å, º)

**Table:**

*D*—H···*A*	*D*—H	H···*A*	*D*···*A*	*D*—H···*A*
N1—H1···S2*B*^i^	0.88	2.64	3.466 (5)	158
N2—H2···N3	0.88	2.32	3.174 (7)	165
N2*B*—H2*L*···N3*B*	0.88	2.43	3.286 (13)	164
N1*B*—H1*B*···S1	0.88	2.55	3.350 (13)	151
N2*C*—H2*M*···N3*B*	0.88	2.50	3.24 (2)	142
N1*C*—H1*C*···S1	0.88	2.58	3.34 (2)	146

Symmetry code: (i) *x*+1, *y*, *z*.
