# Supplementary figures and images for: Crystal structure of [1,3-bis­(di­phenyl­phosphan­yl)propane-κ2 P,P′](N,N′-di­methyl­thio­urea-κS)(thio­cyanato-κN)copper(I)
Source: Acta Crystallogr E Crystallogr Commun. 2015 Feb 11;71(Pt 3):m61–2. doi: 10.1107/S2056989015002479 (PMC4350702; doi:10.1107/S2056989015002479)

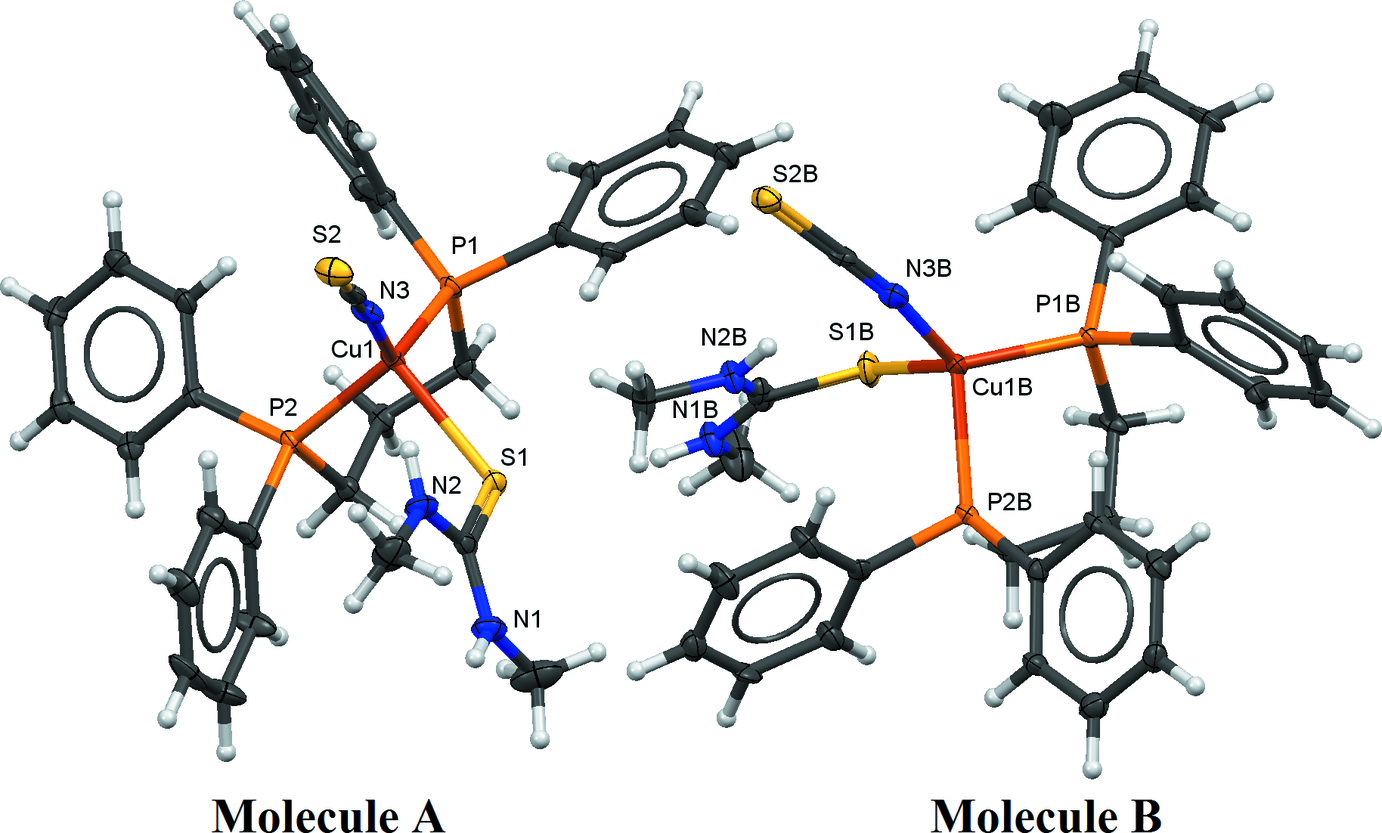

Supplement: Supplementary file 3 [file e-71-00m61-fig1.tif]

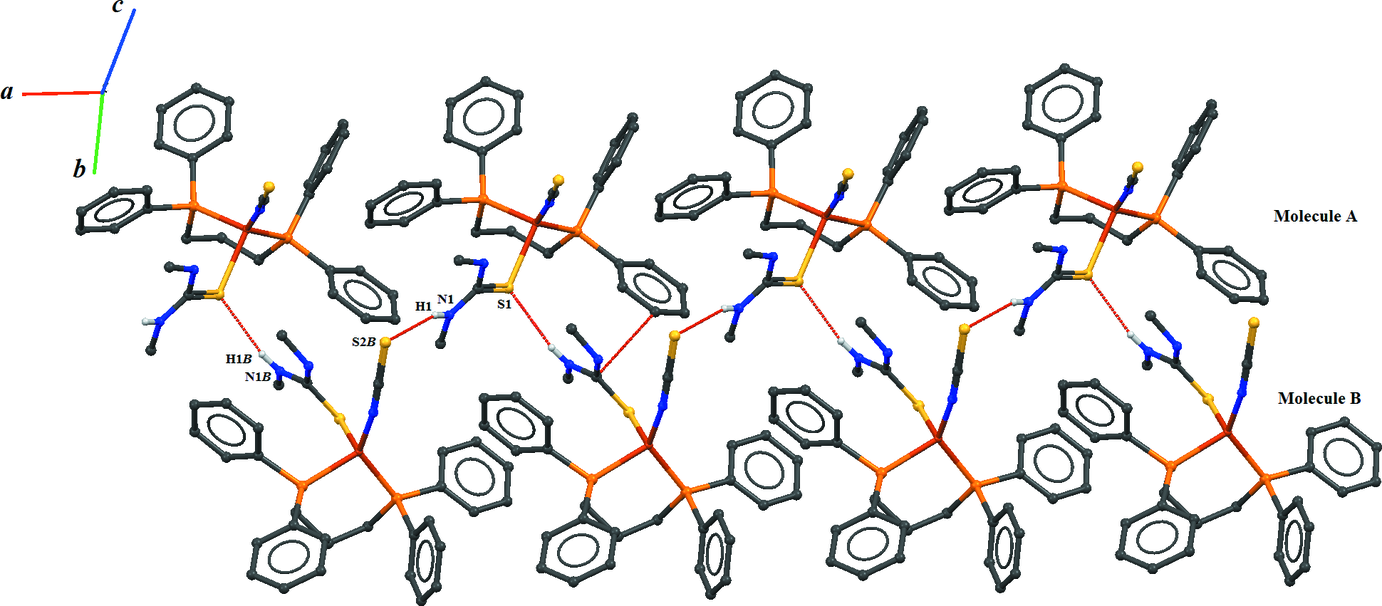

Supplement: Supplementary file 4 [file e-71-00m61-fig2.tif]
